# Evaluating the Quality of Optimal MRCP Image Using RT-2D-Compressed SENSE(CS)Turbo Spin Echo: Comparing Respiratory Triggering(RT)-2D-SENSE Turbo Spin Echo and Breath Hold-2D-Single-Shot Turbo Spin Echo

**DOI:** 10.3390/tomography8030111

**Published:** 2022-05-22

**Authors:** Eun-Hoe Goo, Sung-Soo Kim

**Affiliations:** 1 Department of Radiological Science, Cheongju University, Cheongju 28503, Korea; gooeh@cju.ac.kr; 2 Department of Health Administration and Healthcare, Cheongju University, Cheongju 28503, Korea

**Keywords:** MRCP, compressed SENSE, SENSE, SSh TSE

## Abstract

This study aimed to select the pulse sequence providing the optimal MRCP image quality by applying various reduction and denoising level parameters—which could improve image quality and shorten examination time—to BH-2D-SSh TSE, RT- 2D-SENSE TSE, and RT-2D-Compressed SENSE(CS) TSE and then comparing and analyzing the obtained images. This study was carried out using 30 subjects (15 men and 15 women with a mean age of 53 ± 8.76 years) who underwent an MRCP test using 3.0T MRI equipment. These 30 subjects were composed of 20 patients (CHDD: 7; LC: 6; and IPMN: 7) and 10 volunteers without a disease. When the CS technique was used, five reduction values (1.1, 1.2, 1.3, 1.4, and 1.5) were used and four denoising levels (No, Weak, Medium, and Strong) were used. The existing SENSE method was based on a reduction value of 1, and other parameters were set the same. The image data of BH-2D-SSh TSE, RT-2D-SENSE TSE, and RT-CS-2D TSE used for the analysis were acquired in the coronal plane, and the acquired data underwent MIP post-processing for analysis. To compare these techniques, SNR and CNR were measured for six biliary duct images for the purpose of quantitative analysis, and qualitative analysis was performed on the sharpness of the duct, the overall quality of the image, and the motion artifact. The results of the quantitative and standard analyses showed that the RT-2D-CS TSE technique had the highest results for all IPMN, LC, and CHDD diseases (*p* < 0.05). Moreover, SNR and CNR were the highest when the reduction value was set to 1.3 and the denoising level was set to medium as the CS setting values (*p* < 0.05). Compared with the conventional RT-2D-SENSE TSE, the test time decreased by 20% and SNR and CNR increased by 14% on average. When conducting RT-2D-CS TSE, we found that it shortened the examination time and improved the image quality compared to the existing RT-2D-SENSE TSE. Unlike previous studies, this study using the RT technique shows that it is a useful MRI Pulse Sequence technique able to replace the BH-2D-SSh TSE and BH-3D-SENSE GRASE techniques, which require the patient to hold their breath during the test.

## 1. Introduction

**Figure 1:**
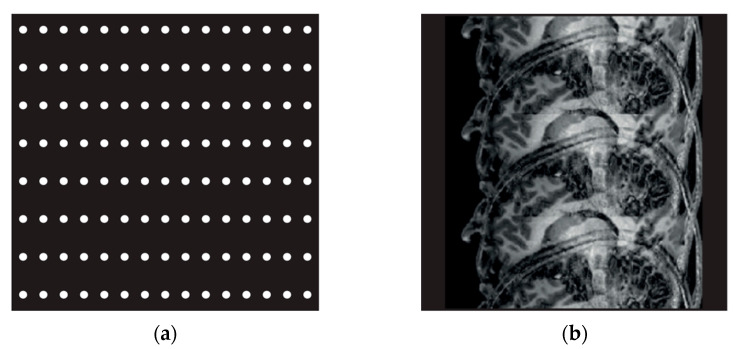
SENSE Techniques. Parallel Imaging (**a**), Aliasing Artifact (**b**).

Magnetic resonance cholangiopancreatography (MRCP) is widely used because it is non-invasive and has no risk of complications as it does not use a contrast medium, unlike conventional invasive tests such as endoscopic retrograde cholangiopancreatography (ERCP) and percutaneous transhepatic cholangioscopy (PTCS) [1,2,3,4,5]. Reducing the time required for acquiring magnetic resonance imaging (MRI) remains an ongoing challenge. In the early 1990s, the parallel MRI acquisition technique based on multiple coil acquisitions emerged as a powerful way to enable faster image acquisition for less MRI acquisition time and better spatial resolution [6,7]. SENSE (sensitivity encoding) provided by Philips (Royal Philips Electronics N. V, Netherlands) is a representative method and widely used. Since MRI images are under-sampled in the SENSE technique, they are reconstructed on K-space data (the 2D or 3D Fourier transform of the image). This reduces the required time, improves resolution, increases the scan range, and enhances the image contrast because it skips the phase encoding step and fills K-space. However, it can cause aliasing artifact (Figure 1) [8]. The aliasing artifact produced in Figure 1b is a phenomenon that takes place since the signals of the receiving coils at the left and right directions overlap as the field of view (FoV) becomes smaller if the images are obtained with the SENSE method.

The image reconstruction equation of SENSE (Equation (1)) can be expressed as follows:

(1) $$p=\underset{p}{\min}\left(\overset{\#\mathrm{coils}}{\underset{i=1}{\sum }}\|m_{d,i}-{\mathrm{ES}}_{d,i}p{\|}_{2}^{2}+\lambda_{1}\|R^{-1/2}p_{2}^{2}\|\right)$$

p: the image to be reconstructed, $m_{d,i}$: the measured data for a given coil element after noise decorrelation, E: the under-sampled Fourier operator as defined by the sampling pattern, $S_{d,i}$: the coil sensitivity for a given coil element after noise decorrelation, obtained with a SENSE reference scan, $\lambda_{1}$: the regularization factor for balancing between data consistency and prior knowledge of the image content. R: coarse resolution data from the integrated body coil obtained from the SENSE reference scan. It is used to constrain the solution during the regularization process [8].

**Figure 2:**
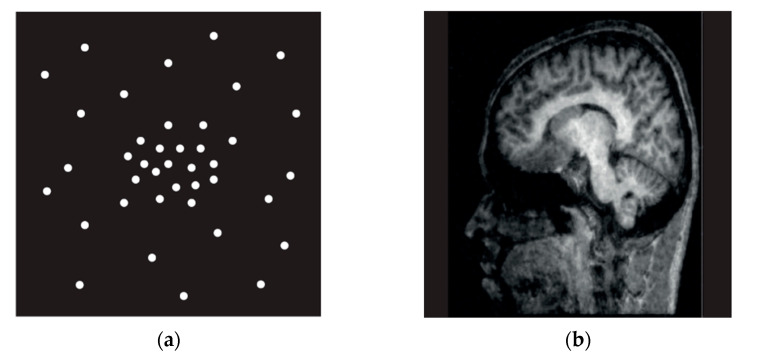
Compressed SENSE Technique. Variable Density Incoherent Under-Sampling (**a**), Noise- Like Incoherent Artifact (**b**).

Compressed SENSE (CS), a more recent technique provided by Philips (Royal Philips Electronics N. V, Amsterdam, the Netherlands), reduces the image acquisition time by under-sampling and overcomes the limitations of the existing parallel images by using the method of erasing noise from sources such as incoherent artifacts, rather than aliasing artifacts that occur in the existing SENSE technique (Figure 2) [9]. Figure 2b illustrates a phenomenon taking place in K-space; since the edge is low and the central part is high for the sampling density, the central part of the image is bright while the edge is dark. This data acquisition method is characterized by its ability to obtain high SNR and a sharp image in the qualitative aspect of the image in a short inspection time.

The image reconstruction equation (Equation (2)) to which CS is applied using these benefits can be expressed as follows.

(2) $$p=\underset{p}{\min}\left(\overset{\#\mathrm{coils}}{\underset{i=1}{\sum }}\|m_{d,i}-{\mathrm{ES}}_{d,i}p{\|}_{2}^{2}+\lambda_{1}\|R^{-1/2}p_{2}^{2}\|+\lambda_{2}\|\mathrm{\Psi p}{\|}_{1}\right)$$

$\lambda_{2}$: regularization factor to balance the sparsity constraining and data consistency in the iterative solution, Ψ: sparsity transform into the wavelet domain [8].

The external reduction factor can be determined by a user, and it indicates how much image acquisition time will be accelerated when applying CS. The value of the factor ranges from 1 to 32. A higher value shortens the time and decreases the resolution. The denoising levels can be divided into “No”, “Weak”, “Medium”, and “Strong”. The user can obtain images in a preferred form by selecting the desired level, and the user can acquire images with a different amount of noise depending on the denoising level. In addition to the previously explained sampling method, various pulse sequences have been developed for quickly acquiring images, such as the SS TSE pulse sequence applied in this study.

The single-shot turbo spin echo (SSh TSE) technique is the same kind of echo as the half-Fourier single-shot turbo spin-echo (HASTE) technique of SIEMENS, and this method acquires all the data of K-Space with a single excitation [10]. It is possible to acquire images quickly by applying a very large TSE factor (=Echo Train Length) value, which is up to 256. This technique also uses the partial Fourier method that obtains data for more than half of the K-space using the mirror-image characteristic and estimates the remaining data. Using this method also played a role in the providing of information on the quality of images with fewer artifacts for an abdominal examination using the breath-hold technique.

A previously examined B-H(breath-hold)-2D TSE MRCP applying the SENSE technique has been conducted [11], and it was also reported that it improved the spatial resolution and increased the signal-to-noise ratio [12]. In addition, motion artifacts that occur during a BH-2D-SSh TSE MRCP examination may cause the omission of anatomical structures. Moreover, it may be impossible to proceed with the examination if a patient has a hearing impairment or is too old to hold their breath because the test requires the patient to hold their breath during the examination. Additionally, when acquiring images with thick slabs, the volume averaging effect in the cross-section can hide small stones or anatomical deformations [13]. Thus, this study obtained images, changing the external reduction factor and the parameter value of the denoising level in RT-2D-CS TSE, and then conducted quantitative and qualitative evaluations. In addition, a pulse sequence was chosen, which provided the optimum MRCP image quality of the three techniques, as compared to BH-2D-SSh TSE and RT-2D-SENSE TSE, which were employed previously.

## 2. Materials and Methods

### 2.1. Study Subjects

This study analyzed the data from 20 patients with a disease (Common Bile Duct dilatation: 7, Liver Cancer: 6, and Intraductal Papillary Mucinous Neoplasm: 7) and 10 volunteers without a disease (30 people in total: 15 male and 15 female; mean age = 53 ± 8.8 years, age distribution: 60 s (13.1%), 50 s (25.8%), 40 s (31.5%), 30 s (29.6%) who underwent a MRCP test using the 3.0T MRI machine. The pulse sequence used for analysis went through MIP post-processing (Philips Ingenia 3.0T CX software, Amsterdam, the Netherlands) after obtaining the coronal plane of BH-2D-SSh TSE, RT-2D-SENSE TSE, and RT-2D-CS TSE data. The parameters of the pulse sequence applied in this study are as follows (Table 1).

### 2.2. Test Methods

The Ingenia 3.0T CX and dStream Torso Coil, which were MRI equipment from Philips (Royal Philips Electronics N. V, Netherlands), were used for data collection. RT was used to proceed with the test according to the patient’s breathing. When the RT-2D-SENSE TSE Pulse Sequence technique was applied, the reduction factor was set to 1 and the patient was tested while breathing through the RT function. In the RT-2D-CS TSE technique to which the Compressed SENSE technique was applied, 1.3 was set as the standard external reduction factor among five factors (1.1, 1.2, 1.3, 1.4, and 1.5), where 1.3 was a value that could maintain the optimal SNR while reducing the image acquisition time. The denoising level was set to Medium among values of No, Weak, Medium, and Strong. The BH-2D SSTSE technique was performed using the breath-hold technique eleven times, and images were acquired while patients were holding their breath for 12 s each.

### 2.3. Analysis Methods

All images were analyzed using the digital imaging and communication in medicine (DICOM) file transmitted to the picture achieving communication system (PACS) system (INFINITT Healthcare). The signal-to-noise ratio (SNR) and the contrast to noise ratio (CNR) were measured for quantitative analysis. They were measured after setting the region of interest (ROI; 8 mm^2^) in each of six hepatobiliary ducts (left hepatic duct, right hepatic duct, common hepatic duct, common bile duct, pancreatic duct, and cystic duct) by using the Image J program (Version 1.52p, National Institutes of Health, Bethesda, MD, USA). The SNR was calculated by dividing the measured signal intensity by the mean of the standard deviation (SD) of the background signal intensity of the image (Equation (3)). The SD of the background was calculated by setting a ROI of 800 mm^2^ in each of the four corners (upper left, upper right, lower left, and lower right) to measure the SNR values more accurately. The CNR was calculated by dividing the difference between the signal in the duct area and the signal intensity of the tissue adjacent to it by the mean of the SD of the background signal intensity of the image (Equation (4)).

(3) $$\mathrm{SNR}=\frac{{\mathrm{Signal}}_{\mathrm{tissue}}}{\sigma_{\mathrm{Background}\;\mathrm{Noise}}}$$

(4) $$\mathrm{CNR}=\frac{\left({\mathrm{Signal}}_{\mathrm{tissue}}-{\mathrm{Signal}}_{\mathrm{adjacent}}\right)}{\sigma_{\mathrm{Background}\;\mathrm{Noise}}}$$

Moreover, the images obtained with three MRI pulse sequence types were evaluated by scoring the sharpness of the duct, the overall quality of the image, and the motion artifact (Very Poor = 1 point, Poor = 2 points, Fair = 3 points, Good = 4 Points, and Very Good = 5 points). The sharpness of the duct is considered to be 1 point when there is no change in the local signal intensity of the duct, 3 points when the change in signal intensity is insignificant and the boundary is not clear, and 5 points when the signal intensity change is good and the boundary is clear. The overall quality of an image is considered to be 1 point when the boundary of the duct is not distinguishable and two or fewer ducts are seen; 3 points when the boundary of the duct looks blurry, there are three or fewer ducts, and only a portion of the duct is distinguished; and 5 points when the boundary of the duct is clear and four or more ducts are observed. The motion artifact is considered to be 1 point when the image cannot be analyzed due to severe artifacts, 3 points when there is an artifact, but it does not impede image analysis severely, and 5 points when there is no artifact. In all items, ratings of 2 and 4 points were determined according to the subjective judgment of the evaluator.

### 2.4. Statistical Analysis

A One way RM ANOVA test was used for the quantitative analysis of three MRI pulse sequence types, and a Bonferroni correction method was used for the post-hoc analysis. For qualitative analysis, the image quality was evaluated using a Friedman test, and the Wilcoxon signed-rank (b > a > c) method was also used for the post-hoc analysis of the Friedman test. Cohen’s Kappa coefficient was used to check the degree of agreement between two observers, and the threshold of Cohen’s Kappa coefficient was 0.6, showing a substantial strength of agreement. It was determined significant when p was less than or equal to 0.05. SPSS Software (SPSS 26.0 for Windows, SPSS Inc., Chicago, IL, USA) was used for all statistical analyses.

## 3. Results

### 3.1. Quantitative Analysis

**Figure 3:**
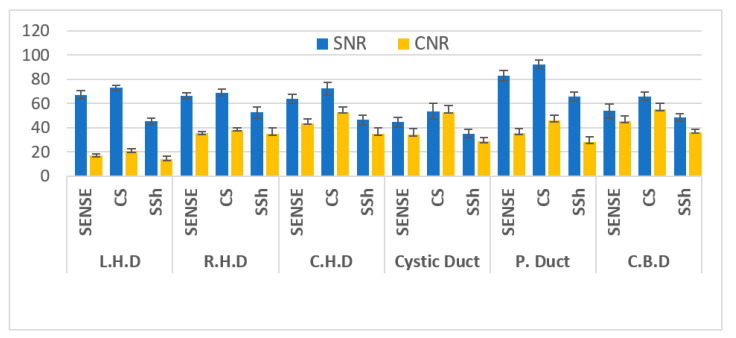
Statistical plots for SNRs and CNRs values in Volunteer’s MRCP Images. SENSE (Sensitivity-Encoded), CS (compressed SENSE), SSh TSE (Single-Shot Turbo Spin Echo).

In the left hepatic duct, right hepatic duct, common hepatic duct, cystic duct, pancreatic duct, and common bile duct images of the 10 volunteers, the SNR measurements of each pulse sequence were significantly (*p* < 0.05) different: BH-2D-SSh TSH < RT-2D-SENSE TSE < RT-2D-CS TSE. The CNR of BH-2D-SSh TSE using the breath-hold technique was the lowest, while that of RT-2D-CS TSE using the RT technique and Compressed SENSE was the highest, just like the SNR measurement (Table 2, Figure 3) (*p* < 0.001).

**Figure 4:**
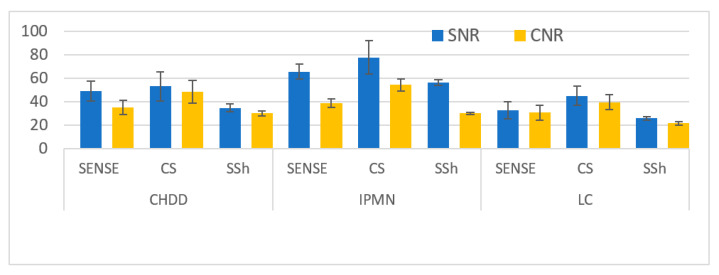
Statistical plots for SNR and CNR values in Patient’s MRCP Images.

The SNR and CNR of common hepatic duct dilatation (CHDD), intraductal papillary mucinous neoplasm (IPMN), and liver cancer (LC) with biliary system lesions showed that LC (30%) was the highest at ≤1 mm, CHDD (35%) was the highest 2 mm or less, and IPMN (35%) was the highest between 1 and 2 mm. The measurements were in the order of BH-2D-SSh TSH < RT-2D-SENSE TSE < RT-2D-CS TSE, similar to the results of healthy people (Table 3, Figure 4) (*p* < 0.05).

### 3.2. Qualitative Analysis

In the TSE Sequence using the BH-2D-SSh, RT-2D-SENSE, and RT-2D-CS techniques, the qualitative evaluation results of the images were significantly (*p* = 0.001) different in the sharpness of the duct, the overall quality of the image, and the motion artifact. In particular, the RT-2D-CS TSE technique obtained high scores of 4.13, 4.46, and 4.56, and it was considered better than the other techniques. In addition, two other techniques showed excellent results with an average of 3.5 or higher (Table 4).

Post-hoc results of Wilcoxon signed-rank test (b > a > c) showed that all three locations were significantly different in RT-2D-CS TSE, RT-2D-SENSE TSE and BH-2D-SSh TSE (*p* < 0.001). The overall quality of an image and motion artifact showed significant differences between all three pulse sequence types (Table 4).

**Figure 5:**
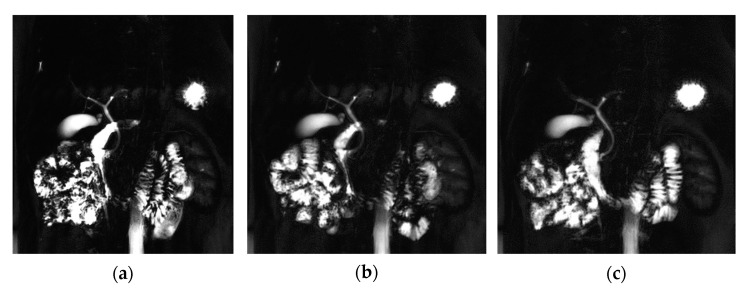
Coronal MRCP Maximum Intensify Projection (MIP) images with RT-2D-CS TSE sequence (**a**), RT-2D-SENSE TSE sequence (**b**), and BH-2D-SSh TSE sequence (**c**) images.

Then, this study examined the differences in MIP images obtained for data analysis. When a, b MIP images were compared between the three techniques in the MRCP images of healthy people, although there was a clear difference in the boundary of the biliary system in terms of the sharpness of the duct, there was no clear difference in the c image. When comparing the signal intensity, the signal was strongly enhanced in the order of a > b > c in the duct lumen. The motion artifact was the strongest in the c image in the order of c > b > a because blurring occurred at the boundary of the biliary tract. In terms of background suppression, blurring in the surrounding area other than the duct was observed in images a and b but not in c. In this respect, the c image acquisition technique was the most effective in background suppression (Figure 5).

**Figure 6:**
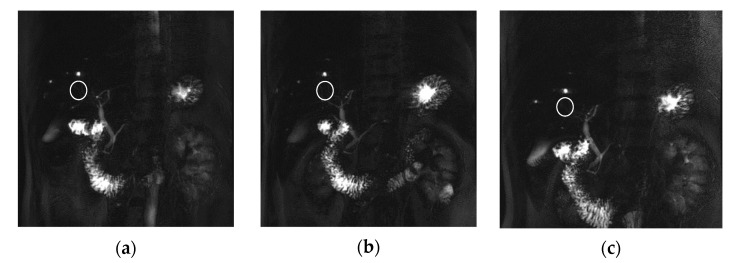
Patient with liver cancer. MRCP MIP images of the RT-2D-CS TSE sequence (**a**), RT-2D-SENSE TSE sequence (**b**), and BH-2D-SSh TSE sequence (**c**) images.

In this study, it also showed that the detection rate of lesions in a certain area was high, such as for the biliary system in the liver area in the MIP image using thin slice thickness. However, the c image acquisition technique is sensitive to breathing, a disadvantage, and if the patient does not cooperate with breathing, the quality of the image will be lowered. Figure 6 showed the images of a hepatic cancer patient, and these biliary system images reveal the signal strength for the disease when comparing three techniques. The signal intensity of the ring-shape duct in the liver area showed a stronger signal strength in the BH-2D-SSh-TSE (c) technique than the other two techniques (a, b; white circle). However, since the noise was high in the background due to the characteristics of the single shot, the SNR and CNR values were not actually evaluated as high. Unlike in previous studies, the analysis result of this study provided new information that the BH-2D-SSh-TSE technique had a higher detection rate for small lesions less than 1 mm.

**Figure 7:**
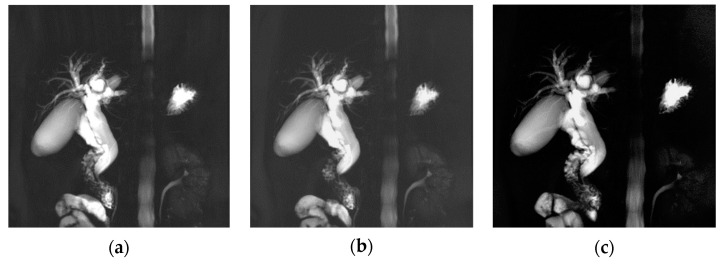
Patient with Common bile duct dilatation. MRCP MIP images obtained with RT-2D-CS TSE sequence (**a**), RT-2D-SENSE TSE sequence (**b**), and BH-2D-SSh TSE sequence (**c**) images.

Figure 7 shows randomly selected images during patient selection; images were acquired without considering breathing. This is an image comparing the three techniques in a patient with CBDD which has been expanded by 2 mm or more in width and length. Unlike in images of diseases that are 1 mm or less, this image visually showed the degree of expansion of CBD and GB, the intrahepatic ducts around the liver, and the overall right bile duct around segments 6, 7, 5, and 8 well. Although the resolution and signal strength were slightly higher in the order of a > b > c, there was no major difference other than that.

## 4. Discussion

The implementation of MRCP in clinical practice can be generally confirmed, depending on whether ERCP is performed and the effect of the procedure, and it is later confirmed by comprehensively synthesizing the location and cause of the lesion [14,15]. Clinically, other than for actual therapeutic and bioptic purposes, MRCP is a groundbreaking technique that provides diagnostic information on the biliary system.

Recently, a method using a parallel imaging technique and a Compressed SENSE technique is emerging clinically as a way to increase spatial resolution, shorten examination time, and compensate for image quality deterioration due to the effect of respiration during abdominal examination [16]. Although previous studies have reported that when Compressed SENSE is applied, a higher denoising level would theoretically improve SNR and CNR [17,18], the quantitative analysis of the MRCP data in this study revealed that the medium denoising level had a slightly higher SNR and CNR than the strong denoising level. When conducting the RT-2D-CS TSE test based on the RT technique and Compressed SENSE, SNR and CNR were the highest when the reduction value was 1.3 and when the denoising level was Medium. Moreover, the biliary system was measured well in the order of RT-2D-SENSE TSE and then BH-2D-SSh TSE. The results of the quantitative evaluation showed that it reduced the test by 20% and that SNR and CNR increased by 14% on average.

Among the three techniques applied in this study, the BH-2D-SSh-TSE technique has been used in clinical practice as a routine protocol of the 2D MRCP oblique coronal test using a breath-hold The biggest advantage it has is that the background suppression is performed well during the MIP mathematical operation process in the image.

This study applied the RT technique and CS technique, which is able to reduce examination time and increase resolution, and evaluated the results for patients who could not cooperate with breathing. Since a previous study applied RT-2D-MRCP and BH-2D-CS-MRCP techniques to cases where patient cooperation was generally successful, CS-BH-MRCP, applying the breath-hold technique and CS, was better in the overall image quality, duct visualization, artifacts, and background suppression tests using SNR, CNR, and the Likert scale. However, it should be noted that it excluded patients who could not cooperate with breathing control [19]. In comparisons of the common bile duct, the largest of the biliary system tissues, it was 26.68 ± 14.58 and 37.48 ± 20.22 with the existing techniques, while it was 65.97 ± 3.63 and 55.03 ± 5.26 with the present technique, and the mean SNR and CNR values were higher in this study than in the comparisons [20].

**Figure 8:**
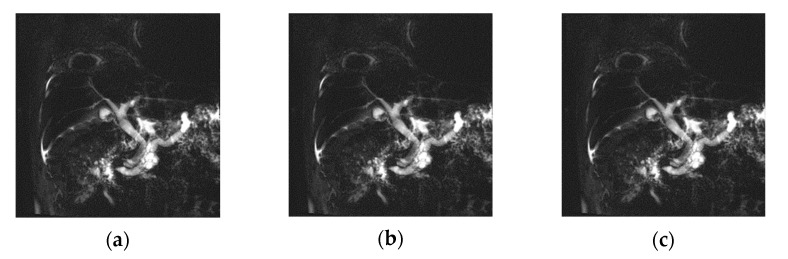
Patient with Intraductal Papillary Mucinous Neoplasm. MRCP MIP images obtained with RT-2D-CS TSE sequence (**a**), RT-2D-SENSE TSE sequence (**b**), and BH-2D-SSh TSE sequence (**c**) images.

A biliary system lesion means an IPMN disease, one of the cystic lesions of the pancreas in addition to the intrahepatic ducts, the anterior branch, and the posterior branch in the liver region. The frequency of MRCP examination has been increasing along with the development of the CS technique, which is a high-resolution technique that was used in the study. Most of these diseases require surgical removal due to their high risk of malignancy [21]. Moreover, based on clinical experience, most patients with these diseases have difficulties in controlling breathing [22]. Figure 8 shows an image obtained from an IPMN patient using the RT method and the B-H method. It is of pancreatic cancer the size of 1–2 mm, and the signal intensity was differed according to the technique. It was confirmed that the main pancreatic duct was dilated, and the signal enhancing effect of the IPMN region was clearly different according to which of the three techniques were implemented. It was a > b > c in terms of resolution and signal intensity, and the boundary area was clear due to the difference in the signal intensity in the diseased area. This technique has been mainly used as an MRCP pulse sequence recently, and the Gradient and spin echo (3D GRASE) MRCP technique using B-H has been effectively applied in clinical practice [23]. The image acquisition principle is to generate a large amount of gradient echo between each turbo spin echo, and it is achieved by switching as the readout gradient polarity is made in the EPI technique.

**Figure 9:**
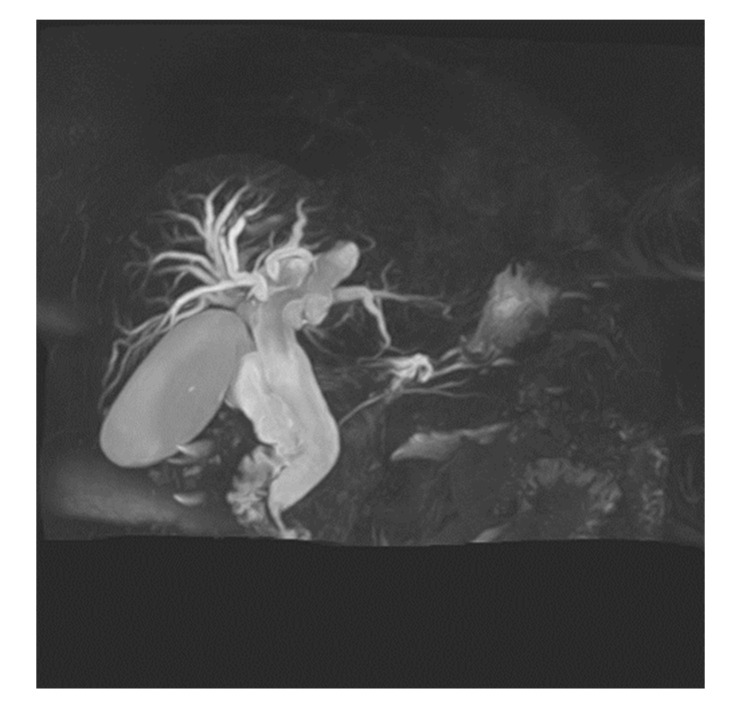
Patient with Common Bile Duct Dilation. MRCP MIP images obtained with a BH-3D-SENSE GRASE sequence. Within a TR, TE of 230, 73 msec, 9 refocusing pulses were applied with 5 gradient-echoes acquired during each echo spacing, resulting in a total scan time of 13 s after parallel imaging acceleration.

The readout gradient polarity is switched, as in the EPI technique. Although this study did not compare it with the other three techniques also used in this study, several cases were obtained to confirm the difference between the B-H technique and the RT technique used in this study. Test parameters were TR = 230 mm, TE = 73, and slice thickness (mm) = 2.4. The test was carried out using the B-H technique after setting FOV (mm), Matrix, and scan time to 350 × 350, 256 × 256, and 13 s, respectively. SENSE was applied as a parallel imaging technique, and the reduction value was set to 2.2, which has been generally used in clinical practice (Figure 9). Since the BH-2D-SSh-TSE technique applied in this study and the BH-3D-SENSE-GRASE technique additionally applied in this study had to be conducted while holding breath, the image quality was inclined to drop drastically if the condition of the patient was poor or it was difficult to communicate with the patient. The image in Figure 9 was acquired using the 3D GRASE technique while the patient with CBIDD held their breath. The patient was in relatively good condition, and the breath-holding time was 13 s. There was no difficulty in the examination.

Since even the image quality of the 3D GRASE technique, which has been widely used in recent years, can be deteriorated depending on the patient’s condition, this study tried to provide for optimal image information by using both RT and CS. One limitation of this study was that it could not compare the SENSE and CS techniques by changing the extra reduction value. However, we believe that this would have been sufficiently compensated for because this study used 1:1.3, which is the correlation between the two factors suggested as the optimal SNR providing the best results by Philips. In addition, there were too few diseases represented by the subjects, and there may be an error found when MIP data, rather than raw data, are measured. However, it could be supplemented for, taking into account the fact that the patients were in good condition.

## 5. Conclusions

When conducting the RT-2D-CS TSE test using the Compressed SENSE, it was possible to shorten the test time and obtain a high-quality image by comparing RT-2D-SENSE TSE and BH-2D-SSh-TSE. Since this method is carried out using RT, there is no need to restrict breathing. Therefore, it reduces the number of artifacts caused by movement. This study concludes that in the future, for patients with a hearing impairment who are unable to hold their breath or for the elderly who have difficulty in holding their breath, this may prove to be a useful MRI Pulse Sequence technique that could replace the BH-2D-SSh TSE and BH-3D-SENSE GRASE techniques that require patients to hold their breath.

## Figures and Tables

**Table 1:** Scanning Parameters for 3 pulse sequences.

Parameters	2D-SENSE TSE	2D-CS TSE	2D-SSh TSE
TR ^a^ (ms)	4093	4093	10696
TE ^b^ (ms)	920	920	920
Slice Thickness (mm)	50	50	50
NEX	1	1	1
FOV (mm)	270 × 270	270 × 270	270 × 270
Matrix	256 × 256	256 × 256	256 × 256
Scan Time (s)	58	46	132
Respiration	RT ^c^	RT ^c^	BH ^d^
SENSE, Reduction	Yes, 1	No, No	No, No
CS, Reduction	No, No	Yes, 1.1, 1.2, 1.3, 1.4, 1.5	No, No
Denoising Level		No, Weak, Medium, Strong	

^a^ Repetition Time, ^b^ Echo Time, ^c^ Respiratory Triggering, ^d^ Breath Hold.

**Table 2:** SNRs and CNRs values in Volunteer’s MRCP Images (*n* = 10, 60 cases).

Location	MRI Sequences	SNR	*p*-Value	CNR	*p*-Value
L.H.D	RT-2D-SENSE ^a^ TSE	67.19 ± 3.34	<0.001	16.84 ± 1.63	<0.001
	RT-2D-CS ^b^ TSE	72.80 ± 2.35	(b > a > c)	20.78 ± 1.92	(b > a > c)
	BH-2D-SSh ^c^ TSE	45.23 ± 2.40		14.02 ± 2.60	
R.H.D	RT-2D-SENSE TSE	66.30 ± 2.26	<0.001	35.52 ± 1.16	<0.115
	RT-2D-CS TSE	68.92 ± 2.86	(a > c, b > c)	38.11 ± 1.88	
	BH-2D-SSh TSE	52.63 ± 4.52		34.80 ± 4.91	
C.H.D	RT-2D-SENSE TSE	63.89 ± 3.61	<0.001	43.73 ± 3.63	<0.001
	RT-2D-CS TSE	72.24 ± 5.23	(b > a > c)	52.95 ± 4.24	(b > a > c)
	BH-2D-SSh TSE	46.43 ± 3.98		34.82 ± 5.07	
Cystic Duct	RT-2D-SENSE TSE	44.57 ± 4.08	<0.001	34.12 ± 4.82	<0.001
	RT-2D-CS TSE	53.50 ± 6.37	(b > a > c)	53.01 ± 5.25	(b > a, b > c)
	BH-2D-SSh TSE	35.21 ± 3.48		28.68 ± 3.07	
P. Duct	RT-2D-SENSE TSE	83.04 ± 4.48	<0.001	35.47 ± 3.89	<0.001
	RT-2D-CS TSE	92.25 ± 3.41	(b > a > c)	46.01 ± 4.38	(b > a > c)
	BH-2D-SSh TSE	65.56 ± 3.70		28.09 ± 4.47	
C.B.D	RT-2D-SENSE TSE	53.76 ± 5.73	<0.001	45.26 ± 4.14	<0.001
	RT-2D-CS TSE	65.97 ± 3.63	(b > a, b > c)	55.03 ± 5.26	(b > a > c)
	BH-2D-SSh TSE	48.42 ± 2.87		36.45 ± 2.22	

Numbers: Average values ± standard deviation. ^a^ SENSE; Sensitivity-Encoded, ^b^ CS; Compressed SENSE, ^c^ SSh; Single Shot, *p*-value: One way Repeated Measure ANOVA, Post-hoc: Bonferroni.

**Table 3:** SNRs and CNRs values in Patient’s MRCP Images (*n* = 20).

Location	2D SENSE ^a^ TSE	2D CS ^b^ TSE	2D SSh ^c^ TSE	*p*-Value
CHDD (*n* = 7) ≥ 2 mm SNR and CNR	48.82 ± 8.29 35.07 ± 6.11	52.93 ± 12.47 48.24 ± 9.55	34.60 ± 3.44 29.82 ± 2.18	<0.001 <0.001
IPMN (*n* = 7) 1 ≤ 2 mm SNR and CNR	65.85 ± 6.11 38.62 ± 3.52	77.71± 14.26 54.12 ± 5.19	56.09 ± 2.22 30.05 ± 0.91	<0.001 <0.001
LC (*n* = 6) ≤ 1 mm SNR and CNR	32.64 ± 7.44 30.43 ± 6.42	44.91 ± 8.13 39.39 ± 6.47	25.56 ± 1.67 21.35 ± 1.35	<0.001 <0.001

Numbers: Average values ± standard deviation. Significant Differences, *p* < 0.001, Post-hoc: Bonferroni b > a > c.

**Table 4:** Subjective grading of sharpness of duct, overall image quality, and motion artifacts in qualitative assessment (*n* = 30).

Location	RT-2D-SENSE ^a^ TSE	RT-2D-CS ^b^ TSE	BH-2D-SSh ^c^ TSE	*p*-Value
Sharpness of duct	3 (2–5)	4 (2–5)	4 (2–5)	<0.001
Overall image quality	3 (2–5)	4 (2–5)	5 (2–5)	<0.001
Moving artifact	3 (2–5)	4 (3–5)	4 (4–5)	<0.001

Note: Numbers are mean ± standard deviation, *p*-value: Friedman test, Post-hoc: Wilcoxon signed-rank test (b > a > c).

## Data Availability

All the data presented in this study are available upon request from the first author.
